# Design and Implementation of e-Health System Based on Semantic Sensor Network Using IETF YANG

**DOI:** 10.3390/s18020629

**Published:** 2018-02-20

**Authors:** Wenquan Jin, Do Hyeun Kim

**Affiliations:** Department of Computer Engineering, Jeju National University, Jeju 63243, Korea; wenquan.jin@jejunu.ac.kr

**Keywords:** healthcare, e-Health, semantic sensor network, IETF YANG, Open Connectivity Foundation IoTivity

## Abstract

Recently, healthcare services can be delivered effectively to patients anytime and anywhere using e-Health systems. e-Health systems are developed through Information and Communication Technologies (ICT) that involve sensors, mobiles, and web-based applications for the delivery of healthcare services and information. Remote healthcare is an important purpose of the e-Health system. Usually, the eHealth system includes heterogeneous sensors from diverse manufacturers producing data in different formats. Device interoperability and data normalization is a challenging task that needs research attention. Several solutions are proposed in the literature based on manual interpretation through explicit programming. However, programmatically implementing the interpretation of the data sender and data receiver in the e-Health system for the data transmission is counterproductive as modification will be required for each new device added into the system. In this paper, an e-Health system with the Semantic Sensor Network (SSN) is proposed to address the device interoperability issue. In the proposed system, we have used IETF YANG for modeling the semantic e-Health data to represent the information of e-Health sensors. This modeling scheme helps in provisioning semantic interoperability between devices and expressing the sensing data in a user-friendly manner. For this purpose, we have developed an ontology for e-Health data that supports different styles of data formats. The ontology is defined in YANG for provisioning semantic interpretation of sensing data in the system by constructing meta-models of e-Health sensors. The proposed approach assists in the auto-configuration of eHealth sensors and querying the sensor network with semantic interoperability support for the e-Health system.

## 1. Introduction

Healthcare is among the fastest growing business domains in the world. It is also one of the most important markets for many countries and districts. This is a big service industry that is consumed by everyone in the world and give jobs to millions of people. According to the report of the World Health Organization (WHO), the world’s population is aging, and the proportion of elderly people is growing faster [1,2]. Elderly care has a high social cost and also results in new challenges to the current healthcare system [3]. Besides taking care of elderly persons, the general healthcare system needs to be improved for monitoring the health status of every citizen [4]. It is very important to identify a disease in the initial stages to diagnostic and treatment more effective and successful. However, most people are very busy with work and social activities in modern society. Therefore, they usually cannot spend the time to visit doctors to check their health status without extraordinary health problems. An eHealth system based on Internet and Communication Technologies (ICT) can be the solution to deliver healthcare services to the people in their daily lives.

The healthcare industry is adopting ICT-based solutions to enhance its efficiency and quality of services. These technologies can provide low-cost health services to improve the living quality of human beings. With advances in ICT, many healthcare applications are being developed that can provide services to support chronic diseases, early diagnosis, real-time monitoring, and medical emergencies through the Internet [5]. Moreover, the technologies of Internet of Things (IoT) enable heterogeneous devices and applications to interwork together to support seamless and autonomous services [6]. IoT devices are constrained devices with limited computation and communication abilities. These devices can be attached to any daily life object to collect desired data. IoT-based solutions provide remote monitoring and sensing services through communications between entities and thus enabling efficient and comfortable services to end users. Various IoT-based solutions have been successfully developed and deployed for various enterprise systems such as healthcare systems [7], factory management system [8], smart homes, and logistics systems [9].

In the context of healthcare support, the concept of e-Health [10] is a new approach to implement a healthcare system that is based on electronic processes and internet communications. A healthcare system with e-Health elements includes users, devices, and servers [11]. Users can be doctors, nurses, patients, and guardians who need to access the system with permissions through devices such as mobile phones or specific e-Health devices [12]. Devices include various sensors attached to the patients for monitoring health status, retrieving health history, and sensing data. Sensing devices are connected to the internet to communicate with the server. In the e-Health system, devices should be portable and easy to operate. The server is the most important element of an e-Health system. It includes the service provider, health data, management, and analysis functionalities for interacting with professionals and patients.

IoT technologies enable data exchange between entities through protocols in e-Health systems. The data can be health sensing data, health history, device information, and other user- or domain-specific data. Each interaction in the e-Health system among the devices require a specific data structure to generate corresponding data. Therefore, these interactions need standardized structures to support interoperability between users and devices and devices and the server [13]. Semantic models are based on ontologies that are useful for concept modeling and include two things, i.e., concepts and relationship among the concepts. Normally, the same concepts are expressed using different terms in different regions, languages, organizations, domains, etc. One possible solution could be the development of a standard vocabulary or terminology, but this is not feasible due many other limitations, i.e., standard vocabulary development is time-consuming, there are adaption issues and ambiguity problems etc. Ontology provide a formal specification to express domain knowledge in such a way so that it can easily be shared. Semantic models can define the data format of content in request and response messages that include health information. The semantic model in healthcare systems shall ideally include e-Health terminologies and the relationship of those terminologies from existing semantic models for generating health messages. The message includes one or more sensors’ sensing data. Each sensing data need to be identified by ID or name. Also, the message should include corresponding e-Health terminologies to explain the meaning of the information for the parser in devices or the server based on the semantic model. Thus, the semantic model enables us to express the information of e-Health sensors that are used for representing the corresponding instance of e-Health sensors in the virtual environment to improve the interoperability between users and the e-Health system.

In this paper, we present an e-Health system with the Semantic Sensor Network (SSN). For the interoperability in the e-Health system, a semantic model is developed using the IETF YANG. YANG is a data modeling language that can be used for the configuration of electronic devices such as network devices, IoT devices, and mobile devices [14]. In the proposed e-Health system, the YANG data is used to configure e-Health devices and represent information of the e-Health sensors. Using the YANG-based semantic model, the system can automatically generate sensing data in a specific message format for transferring to the server through the auto-configuration of the e-Health device. The semantic model acts as a protocol between the e-Health device and the e-Health server, which explains the meaning of the message types to the e-Health server. Once the e-Health server receives the message from an e-Health device, the server can parse the message using a specific parser based on the semantic model for the message type. Furthermore, the semantic model as the information of e-Health elements in the system, which can be used for displaying information to the healthcare system users. In order to apply the YANG-base semantic model to the e-Health system for the semantic interoperability, the design and implementation of interaction between the e-Health server, the e-Health client, and the e-Health device are presented.

The rest of the paper is structured as follows: Section 2 reviews the existing solutions for data exchange and formats for the healthcare system. A brief description of IETF YANG is also presented in this section that we used for defining the semantic model along with a discussion on terminologies used in the semantic model from the Open Connectivity Foundation (OCF) specification. Section 3 introduces the architecture of the proposed healthcare system that includes the user, the e-Health server, the e-Health client, and the e-Health device. Through the interaction among these elements, we present the interoperability of the semantic model in this system. Section 4 presents the semantic model of the e-Health sensors for the semantic interoperability of the proposed e-Health system. In this section, we present the semantic model using ontology and also present the actual data using the IETF YANG. Furthermore, we present the methodology of the semantic interpretation and the application scenario for the proposed system. Section 5 presents the implementation details of the e-health system. Section 6 presents the performance evaluation of OCF IoTivity communications for synchronizing the semantic information in the e-Health device and publishes the sensing data that is generated using the semantic model. Finally, we conclude our paper in Section 7.

## 2. Related Works

Healthcare information can be utilized for several purposes such as clinical facts, decisions, activities, and workflows. In order to provide efficient and improved healthcare services based on IoT technologies, standardization for data exchange, communication protocols, further on frameworks, and platforms are important. The challenges for supporting good healthcare services with e-Health elements are the user interface, connectivity, authentication, security of patient data, and efficient interaction with health professionals according to the requirements of users (patients and health professionals) for healthcare services [15]. Several standards and frameworks have been developed to address these issues. For example, the WHO operates projects for supporting strategies for e-Health applications [16]. HL7 and DICOM provide information exchange standards for e-Health systems to integrate easily into communications between devices [17,18,19]. Through the protocols and Application Program Interfaces (APIs) for heterogeneous frameworks, the e-Health devices are able to interact with each other to exchange data. However, those standards depend on the standardization organizations who define the format of messages and sensing data. The presented semantic model can define the format of messages for a specific type. Therefore, the system can define a specific protocol between the e-Health device and the e-Health server through the semantic model. It can also be extended to involve other standard data structures.

Semantic models can be used for several purposes such as the definition of rules, semantic web for presenting information, and using the semantic model to cluster or filter the data [20]. Zhang et al. present the semantic model to define customized medical rules for setting up alarm conditions by monitoring the sensing data from health sensors such as blood pressure, lipid, and heartbeat [21]. Sezer et al. present semantic model for the healthcare system through describing the domain and device information along with defining rules to provide proper services [22]. Thermolia et al. present the semantic model to extract health knowledge to be used for analyzing the patient records and recommending personalized diagnosis and treatment approaches [23]. In this paper, we use the semantic model to present e-Health information and define the rules for generating specific message format to enable interoperability in the e-Health system.

The ontology based on the Web Ontology Language (OWL) provides a formal specification for the semantics of context data [24]. In this paper, we present an IETF YANG–based approach for the modeling the semantic model. The YANG data modeling language is a data modeling language that is used for configuration and manipulation. YANG language allows data modelers to define the syntax and semantics of device configurations, generating messages for transmission. All YANG definitions are contained in modules. An individual module is identified by its name and has its own XML namespace. A module can be further subdivided into submodules to simplify the maintenance of complex modules. YANG stores all data in the leaf elements of an XML tree. The leaf statement defines a simple leaf carrying a single value. A list of simple leafs, each carrying a single value, can be defined using the leaf-list statement. For implementations of the YANG parser and validator, several commercial and open-source implementations have been developed. For developing an e-Health system, a YANG parser library is needed. CESNET’s libyang is a library (implemented in C language) to support BSD 3-Clouse license and YIN data format parsing and XML translating etc. [25]. The Open Day Light project has developed a Java-based implementation of the YANG library [26]. It also supports XML translating functionalities based on RFC 6020 along with support for translating JSON data. For Python implementations a good example is the NCClient API [27]. There are no such libraries available for developing an e-Health system. Therefore, we have developed our own approach for YANG data processing and interpretation in the proposed e-Health system.

For the interoperability in the SSN-based healthcare system, members of the W3C Health Care and Life Sciences Interest Group (HCLS IG) have published a variety of genomic and drug-related datasets as Resource Description Framework (RDF) triples [28]. Many semantic systems refer the ontology model from the SSN ontology that is published by W3C [29,30]. Ontology development is a time-consuming and a costly process and requires careful deliberation. Instead of developing a new vocabulary for defining concepts in a particular domain, existing terminologies can be time-saving and cost-effective while developing a new ontology [31]. Therefore, many ontologies are built using existing domain terminologies. For instance, the Semantic Sensor Network (SSN) ontology was developed to describe sensors, actuator, sensing data, feature of interest etc. Future ontologies shall ideally extend existing domain ontologies and thus enabling a standard ontology development for complete domain knowledge with the passage of time. In the smart healthcare domain, we need a semantic interoperability platform based on semantic web technologies for standardized healthcare information exchanges between heterogeneous Electronic Healthcare Records (EHRs) in different care settings. Aime et al. have developed a semantic interoperability platform for healthcare information exchange based on centrally managed common data elements (CDEs) [32]. CDEs are linked to various international and national reference terminologies e.g., CCAM, LOINC, PathLex, ICD-O, etc. Therefore, for any specific domain of industry, we can define our own semantic model using specific terminologies. In this paper, we define the semantic model using the IETF YANG. Therefore, the terminologies of the YANG modeling language need to be included. Furthermore, the relationships of the semantic model are also influenced by the IETF YANG.

We refer the terminologies from OCF healthcare-related resources to define the semantic model for our proposed system [33]. In the OCF specification, the resource models are mapped to protocols to enable communications between OCF devices [34]. In addition, the resource models support the semantic information that is required for the interoperability in the OCF network. In the OCF specification, the resource models are defined in the RESTful API Modeling Language (RAML) definition with JSON schemas to represent Create, Retrieve, Update, Delete, Notify (CRUDN) operations. Furthermore, as the OCF specification mentions, those API modeling frameworks can be used for the semantic description, such as RAML and Swagger [35,36]. As shown in Table 1, the resources of the body temperature sensor, blood pressure sensor, electromyography sensor, and galvanic skin response sensor are described that are used in the proposed e-Health system. Furthermore, the ontology of the semantic model involves instances of those sensors.

## 3. Proposed e-Health System

**Figure 1:**
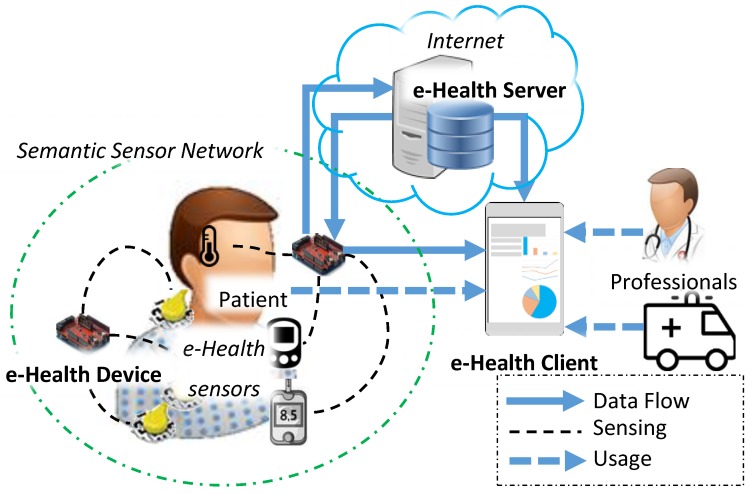
Proposed healthcare system components.

We propose a healthcare system that includes the user, e-Health server, e-Health client, and e-Health device. Figure 1 shows the components of proposed healthcare system. The user can be a healthcare professional or patient who uses the e-Health client to consume healthcare services. The e-Health server is used for supporting services such as monitoring, data saving, professional healthcare services, etc. The e-Health server needs to include a database for saving information such as user information and health data. The e-Health client is used for interacting with the e-Health server and e-Health device. The user can control the e-Health device through the e-Health client that is provided by the e-Health server. The e-Health device can be an IoT device that works in a constrained environment such as low power, small memory devices. The device enables several sensors to be set up for getting health status data from patients.

In the healthcare system, the SSN is deployed around the patient user. The e-Health sensors collect data from the patients and send to the e-Health client and e-Health server. The e-Health sensors are connected to the e-Health device in the personal network. Once the sensing task is triggered by the user using the e-Health client, the e-Health device collects the sensing data from the e-Health sensor that is requested by the user. Then, the user can request the e-Health device to upload the sensing data to the e-Health server. The e-Health device generates the transmission message for the sensing data of e-Health sensors based on the semantic model that is deployed on the e-Health device. Therefore, in the server side, the format of message is interpretable through the semantic model.

The IoT technologies enable healthcare services to be delivered through mobile clients that can be used for displaying user-specific health information and controlling personal e-Health equipment [37]. Based on the mobile client, the private and personalized healthcare service can be provided through the combination of various e-Health sensors in the personal sensor network [38]. The e-Health client is a kind of mobile client that presents the information of sensing data and e-Health sensors to the users in the proposed healthcare system. The e-Health client requests the sensing data from e-Health server and e-Health device. E-Health server also keeps the history data regarding patient health status uploaded by the e-Health device. From the e-Health device, the current health status data is collected and prepared to be uploaded to the server. The information of e-Health sensors is presented based on the semantic model requested by the e-Health client from the e-Health server. The semantic model describes the information of the e-Health sensors that are deployed on an SSN with the user. The information represents the sensors to the users through the e-Health client. Through the Internet, patients and professionals can access the information of the SSN using the e-Health client. The semantic model in the e-Health server can be updated by the system administrator. Once the user updates the e-Health sensors in the personal network, the semantic model for the e-Health device also needs to be updated. Then, using the e-Health client, the user requests the e-Health device to download the semantic model from e-Health server.

## 4. Semantic e-Health Data in the e-Health System

### 4.1. Semantic e-Health Data Modeling

**Figure 2:**
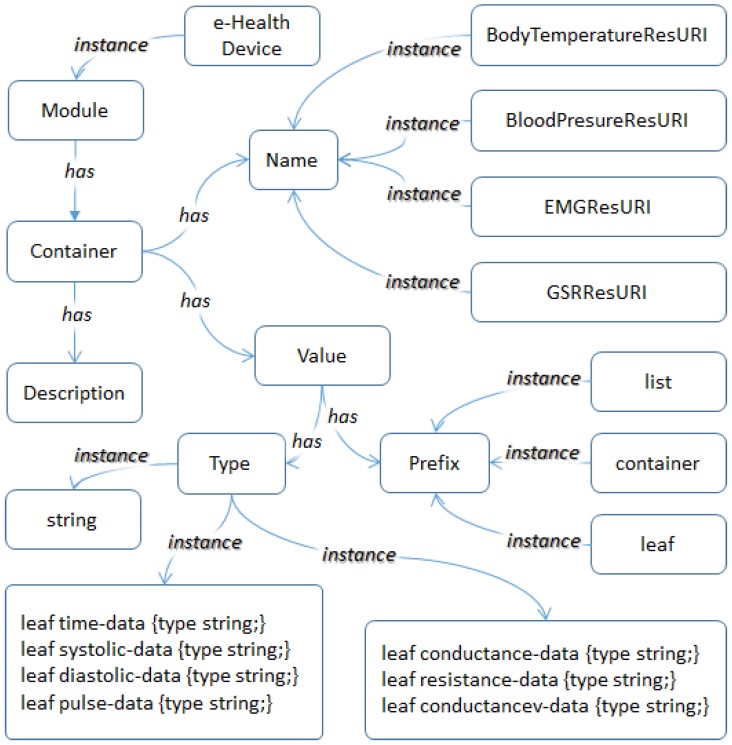
Ontology of the YANG-based semantic model for e-Health sensors.

In the proposed e-Health system, the YANG model is used for the semantic approach. For the YANG-based semantic model, the ontology of the semantic model is presented in Figure 2. The ontology involves the YANG terminologies and information of e-Health sensors in the e-Health system. In order to explain sensors in the e-Health system, the ontology data structures are designed for the YANG data model for the body temperature sensor, blood pressure sensor, electromyography sensor, and galvanic skin response sensor.

In the proposed system, this ontology is used for generating specific message types of sensing data to be collected by the e-Health sensors and presenting the sensors’ information to the e-Health server and clients. As we have adopted the YANG data modeling approach for the proposed system, the ontology includes the terminologies of attributes from the YANG model such as Leaf, Container, and List. For the instance of the Name under the top Container, we present resource URI names of the presented e-Health sensors, which are obtained from OCF.

The root of the structure is Model, and the instance of Module is e-Health device. In the system, only the instance is used. However, if some other brand or type of e-Health device is used, it can then define several instances of Module. After the root, Container has Name, Description, and Value. Container involves the information of an e-Health sensor for an e-Health Device that belongs to a user. A Module can have several Containers to represent a set of sensors that are set up in an e-Health device. Description is used for introducing a Container. It includes some common information about the sensors such as introduction, dimensions, or color. The information is not used for the devices to understand. The users can identify sensors through the semantic information of the e-Health device. Name is used for the identification of a sensor in an e-Health device.

In the proposed e-Health system, we have unique IDs for the identification of sensors, users, and devices in the system. The ontology shows four instances of Name. Each one is a different type of sensor. The identification of a sensor is generated by the system administrator for each type. This means that the identification works on a device scal, because the application of the e-Health device needs to identify sensors through the information. Value has Prefix and Type. Value involves the definition of the message generating mechanism that is used in the application of the e-Health device. In order to use the e-Health sensor kit, Prefix is defined as leaf, container, and list. When a single sensing value needs to be sent, leaf is used with the string type. When multiple sensing types of data need to be sent, container is used with combined type. When a sequential single sensing type of data needs to be sent, list is used with string type. When sequential multiple sensing types of data need to be sent, list is used with combined type.

### 4.2. YANG based Semantic Model for Semantic Interoperability

We present the semantic model using IETF YANG, which defines the semantic model in the YANG data format. According to the YANG modeling principle, the semantic model is designed to involve the terminologies from the YANG modeling language. The proposed model also includes the terminologies of OCF resource specification.

Table 2 shows modules of the YANG model for defining the proposed semantic model. A YANG model can include several modules, and those modules are combined together to compose the semantic model for the e-Health device. We a present body temperature sensor, blood pressure sensor, electromyography sensor, and galvanic skin response sensor in the e-Health device to introduce the usage of the semantic model in this paper. Therefore, BodyTemperatureResURI, BloodPresureResURI, EMGResURI, and GSRResURI are presented for the instances of Name under the Container in the proposed ontology.

For those instances, four types of categories are presented which are leaf, container, list and list with multiple leafs.

- Leaf category is used for generating a message that carries a single value. This module can be used for the blood temperature sensor, which supports a single temperature value.
- Container category is used for generating a message that carries multiple sensing types of data. This module can be used for the blood pressure sensor, which supports sensed time, systolic, diastolic, and pulse in once.
- List category is used for generating a message that carries a sequential sensing values. This module can be used for the electromyogram sensor, which supports a continuous and single type of sensing values in once.
- List with multiple leafs category is used for generating a message that carries a sequential data for multiple sensor outputs. This module can be used for the galvanic skin response sensor, which supports a set of values of conductance, resistance, and conductancev properties.

### 4.3. Methodology of Semantic Interpretation

The proposed semantic model is used for two purposes in the eHealth system. First, the semantic model involves information of the e-Health sensors which are deployed on the e-Health device. The sensors are connected to the Internet through the e-Health device that collects the health data from the user through the sensors and sends it to the e-Health server. Around the user, the sensors compose a sensor network that is connected to the e-Health device and controlled by the e-Health device. The semantic model involves the information of sensor network, and it is deployed on the e-Health device that is downloaded from the server. The information is synchronized in the system to represent e-Health sensors in the cyber world. The meaningful information is presented on the e-Health client that can be used by users such as patients and professionals. Second, the semantic model is used for the data exchange protocol in the proposed system. Many standard solutions are proposed for the data format in healthcare systems. However, some sensors are not defined in the standard data formats. Furthermore, the flexible data formats can involve more cases of sensing functions. In the semantic model, we can define various data formats for sensors. As a protocol, the semantic model defines the specific data format for a sensing data, and the data can be understood in the e-Health system because of the interpretation of parser using the semantic model.

**Figure 3:**
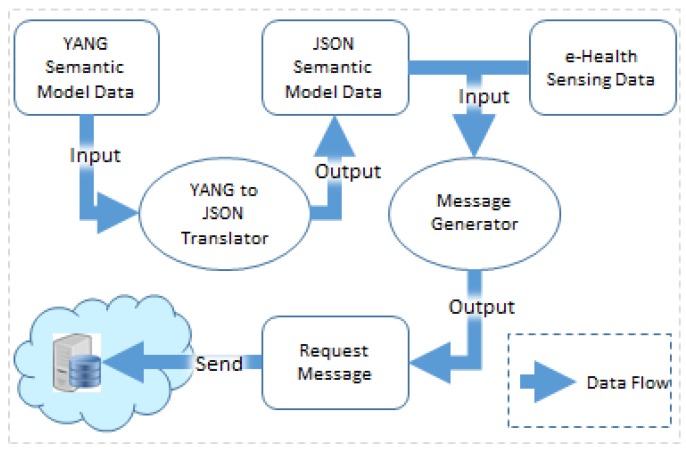
Data flow for semantic interoperability using the proposed semantic model.

For generating the transmission message of sensing data in the e-Health device using the semantic model, the process of semantic interpretation and message generation is needed. Figure 3 shows the data flow to illustrate the mechanism for generating the transmission message of e-Health sensing data using the YANG-based semantic model.

First, the e-Health device needs to request the YANG-based semantic model data from the e-Health server and deploy the e-Health sensors that are connected to the device. In this process, the YANG data is translated to the JSON data using the YANG-to-JSON translator. For the proposed YANG-based semantic model, the system needs a parser to parse the YANG data. However, the YANG parser is not available for the system. Thus, we implement the application to translate YANG to JSON and control JSON data using the existing libraries. However, this approach can be replaced once the appropriate approach is available.

The JSON data for the semantic model which is defined in YANG. The JSON-based data models are generated through the YANG-to-JSON translator. Each YANG container’s value becomes the value of “name” in JSON. The “leaf” statement becomes value of “prefix” in JSON, and the value of “leaf” statement becomes name as “value” in JSON. The “container” statement becomes the value of “prefix” in JSON, and the value of “container” statement becomes name as “value” in JSON. The value of “type” in JSON means that multiple objects are included in for multiple data. The “list” statement becomes the value of “prefix” in JSON, and the value of “list” statement becomes name as “value” in JSON. The “list” statement becomes the value of “prefix” in JSON, and the value of “list” statement becomes name as “value” in JSON. Inside of the “list” block, multiple names for sensing data are defined with the data type. The contents of the “list” in the YANG data are translated to be contents of type in a JSON data.

Once the e-Health device receives the request from the client for sending sensing message, the message generator uses the JSON data and e-Health sensing data to generate the request message for the e-Health sensor. According to the semantic model, the message generator produces the message. Based on the sensor information in the semantic model, the message generator collects the sensing data in different way from the file where the sensing data stored. In this system, four instances enable four ways to read the sensing data file. Based on the four instances of the semantic model, the message generator can recognize the e-Health sensors and the related OCF resource to generate the transmission message in a specific format.

Finally, the e-Health device sends the sensing data to the e-Health server through the Internet.

### 4.4. Application Scenario for Proposed e-Health System

**Figure 4:**
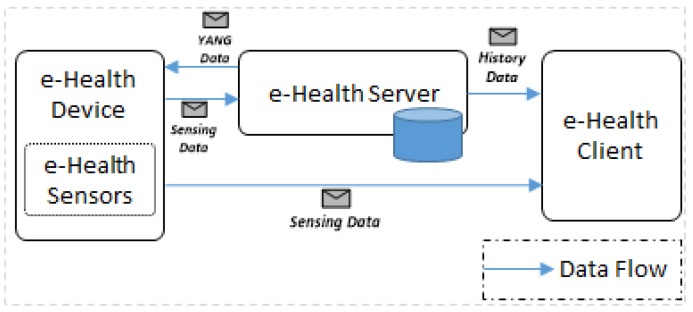
Interaction of components in the e-Health system.

Figure 4 illustrates the interaction of components in the proposed e-Health system. Through the interaction, we present the application scenario that includes activities of initialization, requesting the resource list, requesting sensing data from the device, publishing sensing data to the server, requesting historical data from the server, and requesting the latest sensing data from the device. The key components of e-Health system are an e-Health device with e-Health sensors, an e-Health server with the database, and an e-Health client.

The e-Health client communicates with the e-Health server for retrieving historical sensing data of the e-Health device that is used by the user and communicates with the e-Health device to get the latest sensing data that is stored in the device. The e-Health device communicates with the e-Health server for downloading the semantic model, which is defined in YANG, and publishing the sensing data to store the record of healthcare information.

**Figure 5:**
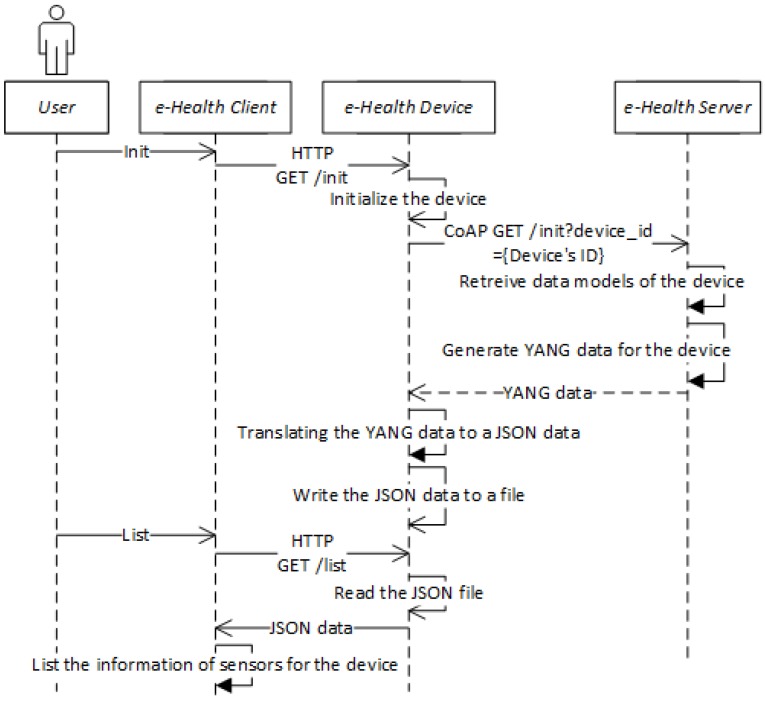
Sequence diagram of the healthcare system for initializing the e-Health device and getting the resource list.

Figure 5 shows the sequence diagram of the healthcare system for initializing the e-Health device and getting the resource list. The Init part of the sequence presents the process in which the user manipulates the client to request the device download the YANG data from the server. The List part of the sequence presents the process in which the user manipulates the client to request the device get the semantic model. The details of those communications are illustrated in the sequence diagram.

**Figure 6:**
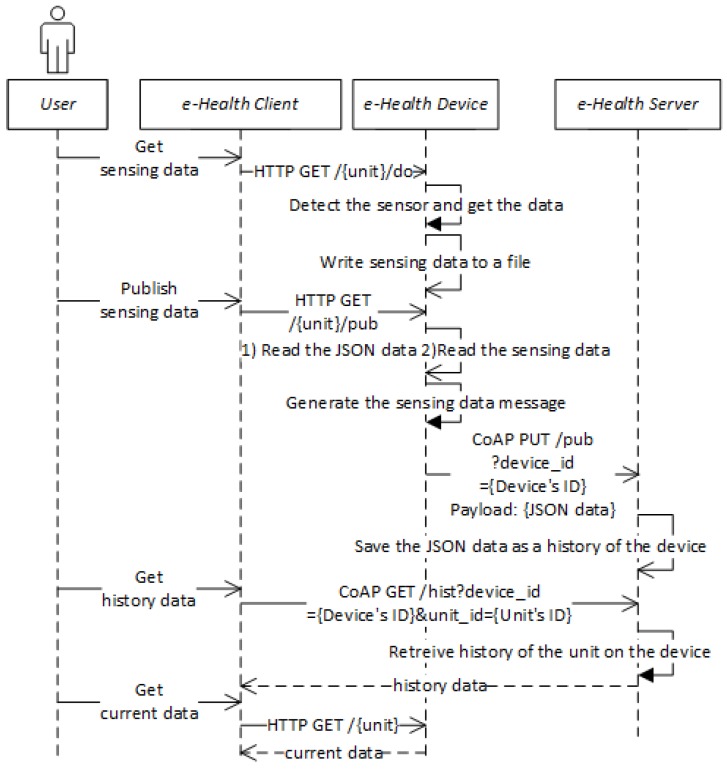
Sequence diagram of the healthcare system for Get sensing data, Publish sensing data, Get history data, and Get current data.

Figure 6 shows the sequence diagram for activities that are activated by the use of e-Health client for the selected e-Health sensor of list. The Get sensing data part of the sequence presents the process in which the client requests to collect the sensing data and store it in the file system. The Publish sensing data part of the sequence presents the process in which the client requests the device publish the collected sensing data based on the generated message. The Get history data part of the sequence presents the process in which the client requests the server retrieve the historical sensing data. The Get current data part of the sequence presents the process in which the client requests the device get the latest sensing data.

## 5. Implementation Details

### 5.1. Implementation Environment

For the developing of the e-Health client, e-Health device, and e-Health server in order to present the proposed semantic approach, we have considered the device, programming language, development tool, library, framework, and DBMS of the e-Health server to implement the system. Each element in the system is developed as a separated project in a specific development environment through the required hardware and software.

Table 3 shows the development environment for the system. The e-Health client project is an Android project that is developed on the Android platform with API level 22. The libraries of the project include the Californium CoAP Framework for communicating with an e-Health server and Volley for communication with the e-Health device. For the e-Health device project, the Intel Edison board–based specific C++ and Arduino project are developed. In order to make a request to the e-Health server through the OCF IoTivity and control GPIO, the libraries of IoTivity 1.0.0 and MRAA 1.1 are included in the C++ project. In the Arduino project, the Wi-Fi library and e-Health library are included for the HTTP server and for controlling the e-Health sensors from the e-Health client. For the e-Health server project, the C++ and C project are developed. The e-Health server includes th SQLite database for saving information and sensing data. In order to provide IoTivity services and CoAP services to e-Health devices and e-Health clients, the libraries of IoTivity 1.0.0 and Libcoap are included.

**Figure 7:**
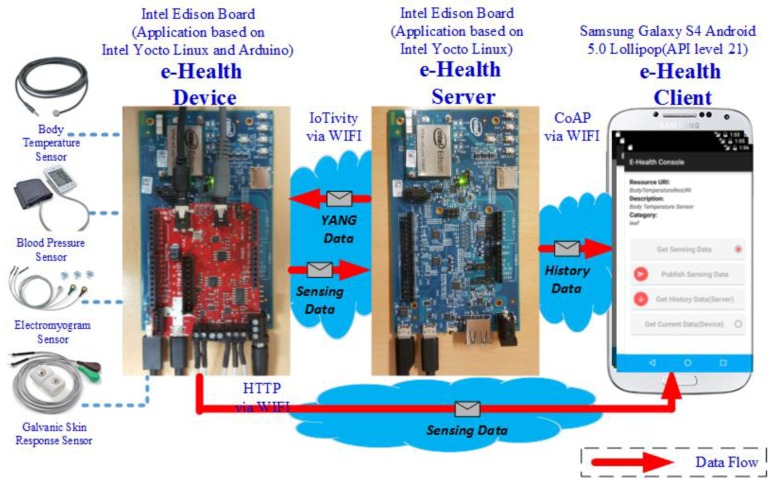
Prototype implementation of the proposed e-Health system.

Figure 7 shows the prototype implementation of the e-Health device, e-Health server, and e-Health client. The e-Health device is equiped with e-Health sensors that are used for the development and are also represented in the semantic model.

A body temperature sensor is used for getting the current human body temperature measured in Celsius. The value of body temperature is calculated as a mean value of continuous sensing data of five times. The blood pressure sensor isused for getting blood pressure-related sensing data. The values are measured once via four types of values, which are time, systolic, diastolic, and pulse. The electromyography sensor is used to get sensing data that related to muscle. The sensor collects continuous data. The galvanic skin response sensor is used to gather emotional. The sensor collects continuous data with conductance and resistance sensing data. The figure also shows the communication flows that have been explained in Section 4.4 for the application scenario of the proposed e-Health system.

### 5.2. Implementation Results

In the e-Health system, the e-Health client requests the data that is saved in the database on the e-Health server. The e-Health client is used for controlling the e-Health device to request the semantic model from the e-Health server and collect the sensing data from the user. The e-Health device includes four e-Health sensors that are used for collecting the user’s sensing data.

Table 4 shows the request message payload for publishing the sensor’s sensing data. Those data are collected from sensors and stored in the file system. Through the mechanism presented in Section 4.3, based on the semantic model, the message generator generates those messages using the sensing data. The message for the body temperature sensor includes the sensing data as a single value because only one value is needed for body temperature. The message for the blood pressure sensor includes time-data, systolic-data, diastolic-data, and pulse-data. The message for the electromyogram sensor includes a series of electromyogram values that are collected by the electromyogram sensor order by time. The message for the GSR sensor includes a series of conductance-data, resistance-data, and conductancev-data values that are continuously collected by the GSR sensor.

**Figure 8:**
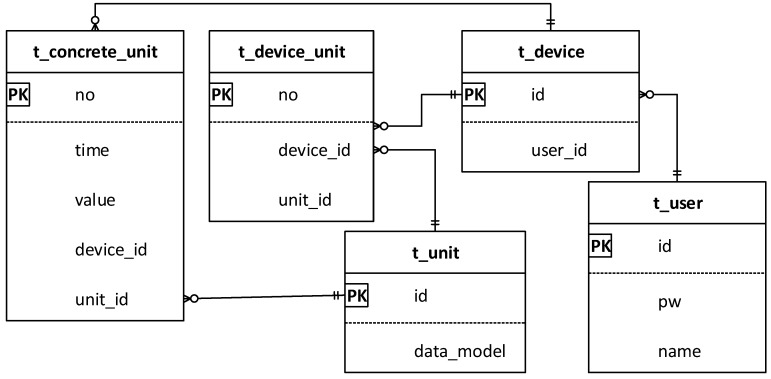
Database ER-diagram of the e-Health system.

For saving the sensor information and sensing data, we present the database of the e-Health server. The database saves the information of the proposed system and also presents the relationship of user, device, and sensor. The database is illustrated in Figure 8 through an ER-diagram. The database of porotype implementation is designed for the least information which is needed in the system.

Table t_user is used for saving user information. This table has columns for id, pw, and name, and those columns are of the varchar type to save string values. The id is the primary used to identify the user. This table has a one-to-many relationship with table t_device, because a user can have several e-Health devices to be used. Therefore, the primary key of t_user is the foreign key of the t_device.

The table t_device is used for saving the e-Health device information that is used by the user. This table has columns for id and user_id, and id is the int type with auto-increment. This table has a one-to-many relationship with table t_concrete_unit and has a many-to-many relationship with table t_unit. Table t_concrete_unit is used for saving sensor information that is installed on the device. The column no is the primary key in int type with auto-increment. The column time is used for saving the creation time of the sensor information. The column value is used for saving sensing data.

The table t_unit is used for saving the type of e-health sensor. The table includes YANG modules for each category of sensors. Using this YANG data with user-specific information, the server generates the user-specific semantic model. The table t_device_unit is used for linking the t_device and t_unit for enhanced query quality because of the many-to-many relationship between the t_device and t_unit.

**Figure 9:**
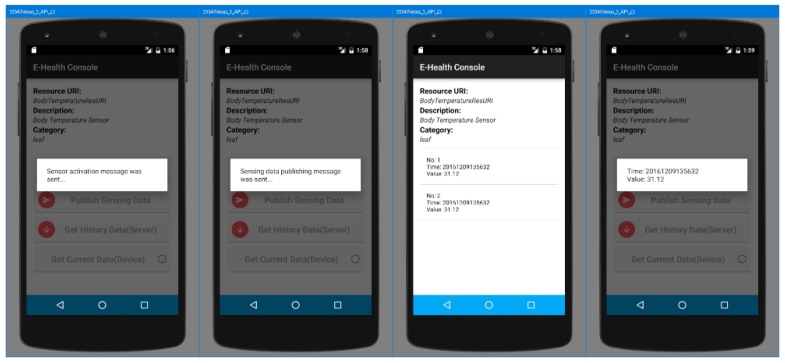
Demonstration screenshots of the e-Health client.

Figure 9 shows screenshots from the e-Health client to present the activities of requesting the sensor to collect the sensing data, requesting the device to publish the sensing data to the server, requesting historical sensing data from the server, and requesting the latest sensing data from the device. Those activities are applied to the body temperature sensor. The client shows the list of sensors that are deployed with the device. Each item in the sensor list includes the sensor resource name, description, and YANG category. In the list, once an item is clicked, the page moves to the page that is used for controlling the sensor.

The page, which is presented once the item is clicked, shows the detail information of the sensor and buttons for interacting with the device and server. The buttons of Get Sensing Data, Publish Sensing Data, Get Current Data are used for interacting with the device, and the button Get History Data is used for interacting with server.

Once the Get Sensing Data button is clicked, the client requests the device collect sensing data through the sensor. Then, the device stores the sensing data on the file system. Each sensor can have a different style for writing the sensing data, as mentioned in Section 4. Once the Publish Sensing Data button is clicked, the device generates the transmission message using the sensing data based on the semantic model. The semantic model enables the message generator to understand the category of sensing data, as presented in Section 4.3. The button Get Current Data is used for requesting the latest sensing data from the device. The message is also generated through the message generator using the semantic model. Therefore, from the screenshot, we can see that the client can present the sensing data in a formed result. The button Get History Data is used for requesting historical data from the server.

Before the list page, there is an Initialization button that is used to download the semantic data from the server. The e-Health device runs the Arduino application to support HTTP services for receiving requests from the e-Health client. The e-Health device needs to download the YANG-based semantic model from the e-Health server. This activity is triggered by the initialization activity.

## 6. Performance Evaluation

In order to estimate the performance of IoTivity communication-based interactions in the presented prototype implementation, we present the Round-Trip Times (RTTs) for activities of Get YANG Data, Send Body Temperature Data, Send Body Pressure Data, Send EMG Data, and Send GSR Data.

In this section, we only present the results for our proposed e-Health system in terms of round-trip time. The experimental setup for other solutions will be completely different. Therefore, a performance comparison cannot be made. These results can be treated as benchmark results for our proposed e-Health system, and other can adopt this solution if it fits their requirement. Table 5 shows the results of calculating the RTTs for each activity. The RTTs are calculated 20 times, and the reported results given in Table 5 present the maximum, minimum, average, and standard deviation for each IoTivity request/response. The prototype implementation is presented through the interactions of the e-Health client, devicem and server. The detailed experimental environment has been presented in Section 5. The communications are based on the IoTivity through the Wi-Fi network. The presented activities are activated by the client, and the communications are conducted between the device and server. Once the device receives the command from the client to start an activity, the device makes requests to the server through the IoTivity. Get YANG Data is the process that is used for downloading the semantic data from the server. Send Body Temperature Data, Send Body Pressure Data, Send EMG Data, and Send GSR Data are used for publishing the sensing data to the server. In this process, the sensing data is generated by the message generator and is sent by the device. Therefore, the RTT is the sum of message generating, parsing, and communication time. The measured results of the RTT values are quite high because of the inherent problem of our current IoTivity-based implementation. In the communication of IoTivity client and server, according to the current version of framework, the client first needs to discover the server. Therefore, all communication includes the on-demand discovery of the IoTivity server before the request is processed. In the future, we look forward to exploring other solutions to address this issue so that server discovery will be done only once.

**Figure 10:**
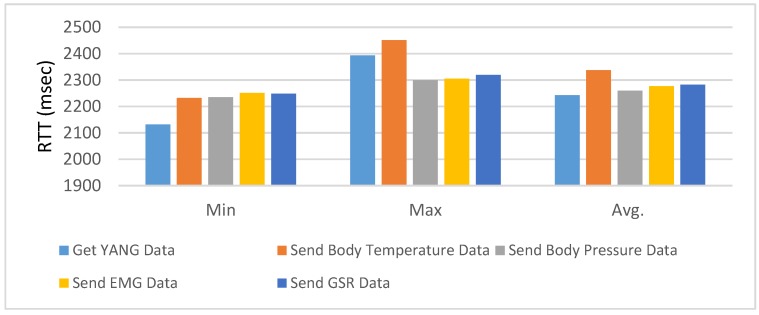
Comparison of RTT for each IoTivity communication of activities.

Figure 10 illustrates the calculated result of RTTs that have been carried out for each function given in Table 5 for better interpretation. The results indicate that the minimum value for Get Yang Data is less as compared to Send Body Pressure Data, Send Body Temperature Data, Send EMG Data, and Send GSR Data. The Send Body Temperature Data have a maximum value as compared to other activities. The average value for Send Body Pressure Data is fewer as compared to other counterpart activities.

## 7. Conclusions

In this paper, we have proposed a semantic model based on the IETF YANG to enable the semantic interoperability in the e-Health system that is comprised of an e-Health device, client, and server. For the prototype implementation, sensors for body temperature, blood pressure, electromyography, and galvanic skin response are used to collect health status data from the user. Furthermore, the semantic model is also designed to include terminologies of OCF that reference those sensors. The sensors have their own specific sensing types that are defined as instances in the semantic model. For those instances, four types of categories are presented: leaf, container, list, and list with multiple leafs. Through the presented categories of instances, heterogeneous semantic models can be designed flexibly to support the semantic approach for various e-Health sensors based on the sensing types such as single value, multiple sensing types of data, sequential sensing values, and sequential data for multiple sensor outputs. For the semantic interpretation, we have presented a solution to generate the transmission message based on the semantic model. Our adopted solution includes YANG-to-JSON translator and message generator, which use the YANG-based semantic model and health sensing data to generate the OCF IoTivity request. The semantic model is also used for presenting the meaningful information of the SSN to the user through the e-Health client. The performance evaluation of proposed semantic approach with the OCF IoTivity communication is estimated in terms of round trip time (RTT). However, the collected results for RTTs are quite high because of the inherent problem in the current IoTivity-based implementation. In the future, we will find an appropriate solution for the IoT protocol to conduct the process rapidly. We are exploring other solutions to address this issue so that server discovery will be done only once. We believe a significant reduction in RRT results can be achieved by incorporating such a solution.

## Figures and Tables

**Table 1:** OCF healthcare resources for e-Health services.

Sensing Title	OCF Healthcare Resource Type	Example URI
Body temperature	oic.r.bodytemperature	/BodyTemperatureResURI
Blood pressure	oic.r.blood.pressure	/BloodPressureResURI
Electromyography	oic.r.emg	/EMGResURI
Galvanic skin response	oic.r.gsr	/GSRResURI

**Table 2:** Semantic model of e-Health sensors based on YANG.

Category	Data Model of e-Health Sensors based on YANG
Leaf	container BodyTemperatureURI { description “Body Temperature Sensor” leaf value {type string;} }
Container	container BloodPressureResURI { description “Blood Pressure Sensor” container value { leaf time-data {type string;} leaf systolic-data {type string;} leaf diastolic-data {type string;} leaf pulse-data {type string;} } }
List	container EMGResURI { description “Electromyogram Sensor” list value { key “data”; leaf data {type string;} } }
List with multiple leafs	container GSRResURI { description “Galvanic Skin Response Sensor” list value { key “conductance-data”; leaf conductance-data {type string;} leaf resistance-data {type string;} leaf conductancev-data {type string;} } }

**Table 3:** Development environment.

Component	e-Health Client	e-Health Device	e-Health Server
Device	Android Phone (API Level 22)	Intel Edison Board (Intel Yocto Linux Image 0606)	Intel Edison Board ( Intel Yocto Linux Image 0606)
Programming Language	Java	C++, Arduino	C++, C
Development Tool	Android Studio 2.1	Eclipse IDE by Intel, Arduino 1.6.1	Eclipse IDE by Intel
Library/Framework	Californium CoAP Framework, Volley	IoTivity 1.0.0, MRAA 1.1, Liberum e-Health Tool Kit Library, WIFI Library	IoTivity 1.0.0, Libcoap

**Table 4:** Request message payload.

Sensor	Payload
Body Temperature Sensor	{"unit-id":"unit001","time":"20160910145418","value":"33.10"}
Blood Pressure Sensor	{"unit-id":"unit002","time":"20160910145427", "value":{"time-data":"201609101433","systolic-data":"132","diastolic-data":"80","pulse-data":"96"}}
Electrocardiogram Sensor	{"unit-id":"unit003","time":"20160910145434", "value":["330.00","390.00","411.00","381.00","399.00"]}
Galvanic Skin Response Sensor	{"unit-id":"unit004","time":"20160910145450", "value":[{"conductance-data":"-1.000000","resistance-data":"-1.000000","conductancev-data":"0.493646"},{"conductance-data":"-1.000000","resistance-data":"-1.000000","conductancev-data":"0.498534"},{"conductance-data":"-1.000000","resistance-data":"-1.000000","conductancev-data":"0.498534"},{"conductance-data":"-1.000000","resistance-data":"14614300.000000","conductancev-data":"0.493646"},{"conductance-data":"-1.000000","resistance-data":"-1.000000","conductancev-data":"0.493646"}]}

**Table 5:** Round trip time (RTT) in milliseconds for each IoTivity communication of activities.

Activity	Min	Max	Average	SD
Get YANG Data	2132	2393	2243	77
Send Body Temperature Data	2232	2451	2337	71
Send Body Pressure Data	2235	2299	2260	18
Send EMG Data	2251	2305	2276	17
Send GSR Data	2248	2319	2282	23
